# Achieving Robust Medical Coding in DRGs Systems: Innovative Actions Adopted in Greece

**DOI:** 10.3390/healthcare12171782

**Published:** 2024-09-06

**Authors:** Charalampos Platis, Leonidas Papaioannou, Panagiota Sideri, Pantelis Messaropoulos, Konstantinos Chalkias, Nikolaos Kontodimopoulos

**Affiliations:** 1 KETEKNY (Greek DRG Institute), 10677 Athens, Greece; ch.platis@instdrg.gr (C.P.); l.papaioannou@instdrg.gr (L.P.); psideri@instdrg.gr (P.S.); p.messaropoulos@instdrg.gr (P.M.); k.chalkias@instdrg.gr (K.C.); 2 School of Social Sciences, Hellenic Open University, 26335 Patras, Greece; 3 Department of Economics and Sustainable Development, Harokopio University, 17676 Athens, Greece

**Keywords:** diagnostic-related group, coding, health systems, efficiency, activity-based funding, innovation, digital platform, artificial intelligence

## Abstract

The purpose of this study is to evaluate and illustrate the effectiveness of a specialized digital platform developed to improve the accuracy of medical coding during the full implementation of Greece’s new DRG system, and to highlight innovative actions for achieving and/or improving accurate medical coding. Already grouped DRG cases recorded in the first DRG implementation year in the region of Crete were examined. A sample of 133,922 cases was analyzed and audited, through a process consisting of three stages: (i) digitalization, (ii) auditor training, and (iii) control and consultation. The results indicated that a significant proportion of DRG coding, with a length of stay exceeding one day, was reclassified into different DRG categories. This reclassification was primarily due to coding errors—such as the omission of secondary diagnoses, exclusion of necessary medical procedures, and the use of less specific codes—rather than mistakes in selecting the principal diagnosis. The study underscores the importance of medical coding control and consulting services. It demonstrates that targeted actions in these areas can significantly enhance the implementation of the DRG coding system. Accurate medical coding is crucial for transparent allocation of resources within hospitals, ensuring that hospital services and reimbursements are appropriately managed and allocated based on the true complexity and needs of patient cases.

## 1. Introduction

Optimizing funding for inpatient care has been crucial for the Greek healthcare system [1]. In 2011, Greece launched a new hospital reimbursement system, KEN, inspired by the DRG (Diagnosis-Related Groups) concept. The primary difference between the KEN and DRG systems is that KEN was based solely on the patient’s main diagnosis, without considering the medical procedure. Furthermore, the KEN classification was conducted manually due to the absence of supporting software. Additionally, the costs associated with KEN were not systematically updated. Limitations arose due to the absence of (i) proper medical classifications suited for a case mix system, (ii) an automated DRG grouping tool, and (iii) a process for cost adjustments. These limitations resulted in the KEN system proving initially inadequate to provide fair and objective budget allocation among hospitals within the health system [2,3,4].

Established by 2014 legislation, the Greek DRG Institute, known as the National Center for Documentation and Costing of Hospital Services, hereinafter referred to as “KETEKNY”, and initially known as ESAN S.A, is an independent, self-governing body with a core mission to implement a fair and transparent system for hospital service costing and reimbursement. KETEKNY’s central goal is to create an integrated system for scientifically based costing and compensation of hospital care in Greece. This approach aims at promoting an evidence-based healthcare system, ensuring that resources are allocated efficiently. KETEKNY initially signed a License Agreement with the German limited liability company InEK GmbH to secure the rights to implement a DRG system in Greece, modeled after the German system. Since then, the Greek DRG system has been updated in two major revisions in 2019 and 2021. KETEKNY, drawing on best practices from around the world, focuses on developing, implementing, monitoring, and updating a Diagnosis-Related Group (DRG) patient classification system.

In January 2021, KETEKNY selected the University General Hospital of Heraklion (PAGNI) to first implement the Greek DRG system due to its wide range of specialties and services. A Joint Ministerial Decision on 7 April 2021 authorized PAGNI to use the Greek DRG coding guidelines for all inpatient cases and update financial data. By 24 December 2021, the DRG system was extended to all hospitals in Crete. Following a further decision on 28 November 2022, hospitals in Macedonia and eight other locations across Greece adopted the system. Finally, the remaining public hospitals and three private entities (Papageorgiou General Hospital, Onassis Cardiac Surgery Center, and Santorini General Hospital) began using the Greek DRG system after a final decision on 21 December 2023.

The implementation of a DRG system in a country raise concerns about the accuracy of medical coding [5]. Medical coding is the conversion of health diagnoses, procedures, medical services, and equipment into internationally common alphanumeric codes. For this reason, it is necessary to check the medical coding in order to document the correctness of the entries and therefore of the data to be evaluated and used [6]. According to the WHO report (2012), the various causes of miscoding can be identified through an effective control mechanism. The causes for errors can be both unethical coding practices (which are the subject of a stronger audit process) and subjective errors in coding practices (which require more intensive training) [7].

The quality of hospital discharge data is crucial for accurate DRG case allocation, as it directly affects how cases are categorized [8]. DRG-based payment systems can potentially reduce the length of stay (LOS) [9], with technological advances like minimally invasive procedures contributing to faster recovery and discharge [10]. Effective implementation requires personnel training, adherence to quality standards, and clear guidelines [11]. Countries such as France have enhanced their DRG systems to better manage hospital activity and ensure appropriate care [12]. To improve hospital efficiency and fairness in payments, it is essential to leverage data for benchmarking, identify efficient providers, and share best practices [13]. Flexible governance and accurate patient classification are keys to the success of DRG-based funding and achieving policy goals [14].

**Figure 1:**
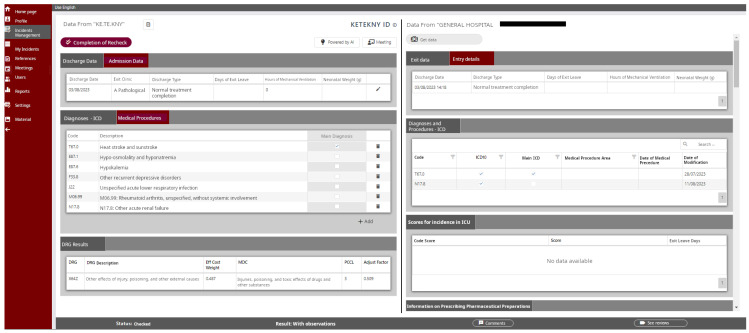
Print screen from the digital platform.

The purpose of this study is to evaluate and illustrate the effectiveness of a specialized digital platform developed by KETEKNY to improve the accuracy of medical coding during the full implementation of Greece’s new DRG system. The platform (Figure 1) was created in response to concerns about coding accuracy, which is crucial for the correct categorization of patient cases and the equitable allocation of hospital resources. By systematically capturing and analyzing medical information from patients’ files in accordance with the Greek Coding Guidelines, the platform aims to identify and correct coding errors. Additionally, it serves as a comprehensive tool for enhancing medical coding practices by providing real-time auditing, enabling targeted training, and facilitating the implementation of new coding rules and guidelines. Through this study, the platform’s role in ensuring accurate, reliable medical coding is assessed, offering insights into strategies that can be adopted to support DRG system integrity and healthcare quality.

## 2. Materials and Methods

The implementation of the project included current situation analysis, assessment of needs, development of a platform, auditing, analysis of results, and training of healthcare professionals in the clinical coding of incidents by the DRG coding system.

### 2.1. Sampling and Audit Process

During the project, the already grouped DRG cases from the first year of implementation in the region of Crete were examined. Due to limited time available for conducting the audits, a sample was drawn and 133,922 cases, out of a total of 200,000, were analyzed and audited. The sample was randomly selected from all hospitals in the region for the first 12 months of the initial year of DRG system implementation. The sampling method used was stratified random sampling, ensuring that the same percentage of total random cases was chosen from each hospital. The audit process consisted of the following stages:

(i) Digitalization: The Greek DRG Institute developed a digital platform to support auditing, which resulted in the establishment and strengthening of medical coding control mechanisms. This intervention aimed to identify and address any discrepancies between the coding of a patient’s medical record and the allocated DRG coding, based on the Greek Coding Guidelines. The platform boasts a highly sophisticated architecture. Data flows seamlessly directly from hospitals, and through secure connections, to KETEKNY’s specialized Grouper software (version 1.0). On a single, user-friendly interface, the platform presents two key data sources: (i) data from the Grouper software, which analyzes and classifies hospital medical coding data collected directly from hospitals’ coders, and (ii) individual patient medical files, which provide comprehensive patient information for a holistic view.

This consolidated view empowers auditors to efficiently conduct controls by having all relevant information readily available on a single screen. Furthermore, the platform offers a range of user-friendly functionalities surrounding this core function, including easy-to-use browsing menus for efficient navigation, the ability to make corrections and add notes for improved clarity, a feature to assign statuses to cases for better organization, and the option to schedule meetings with therapists or physicians for further clarifications.

These features streamline the auditing process and facilitate effective communication with medical personnel, all while maintaining the highest standards of data privacy. The platform is built on a foundation of data anonymization and strict adherence to GDPR regulations at all phases of the auditing process. During the transfer of data from the hospital’s patient files to the auditing platform, the patient’s name and registration number are encrypted, ensuring that no one has access to this information.

(ii) Auditors training: A team of highly skilled auditors underwent specialized training in medical coding according to the DRG classification system, conducted by experts from the Greek DRG Institute.

(iii) Control and consultation: Performed by specialized auditors on a randomly selected sample of cases. The objectives of this process were to verify the accuracy of the classification of incidents in the DRG system and to ensure the completeness of the coding, in accordance with the information obtained from the patient’s medical file. Additionally, the auditors assessed whether the coding adhered to the Greek Coding Guidelines. A key focus was to promptly address issues of under- and over-coding, which are recognized in the international literature and are mitigated through ongoing training for coders. These efforts were designed to enhance and streamline the process of medical recordkeeping, objectively showcase the work performed by each hospital, and promote fair resource allocation within the healthcare system.

The audit platform and the results of the provision of medical coding control and consulting services will be used for the correct implementation of the DRG system by ensuring the quality of medical coding and the documented distribution of available resources in hospitals. These implemented actions can include highly targeted training sessions, for example, focusing on the most frequent mistakes or errors. Additionally, interventions can be made that address both user experience and the medical file itself, potentially including the adoption of new, sophisticated rules or restrictions. Finally, prediction models can be adopted to suggest more accurate codes and schedule periodic controls.

### 2.2. Process Flow and Analysis

**Figure 2:**
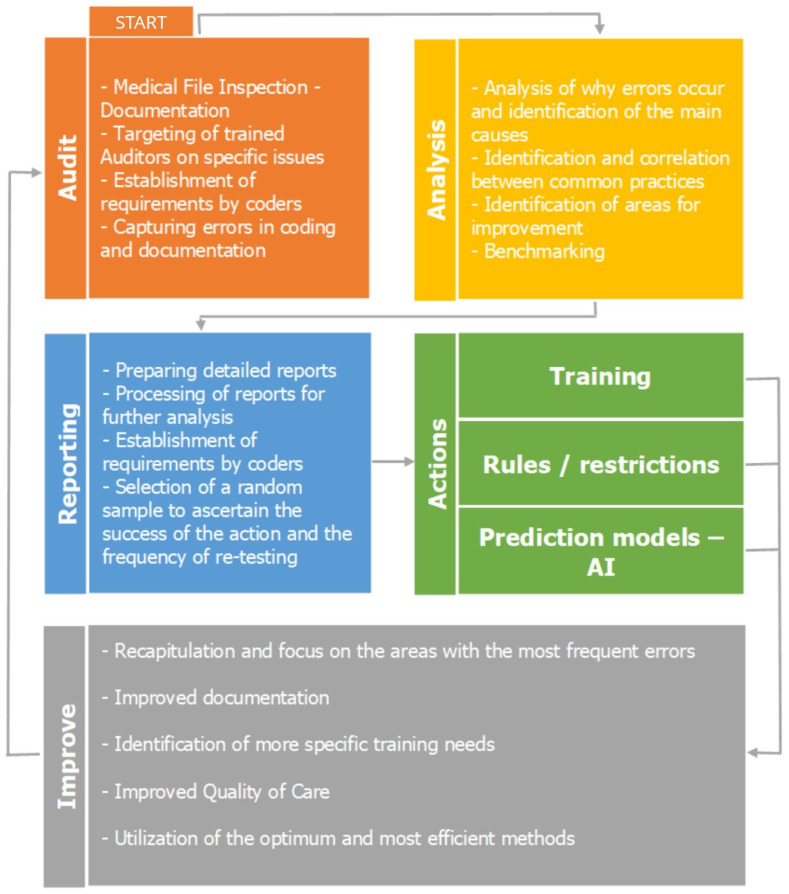
The flow of the total process.

The flowchart in Figure 2 illustrates the flow of the total process. The initial step of the process is the auditing phase, where the auditors proceed with the medical file inspection to capture errors in the hospitals’ coding. In the second stage, the analysis begins with descriptive statistics to identify the reasons why the errors occurred and determine the main causes. This stage identifies the areas that require further improvement. In the next step of reporting, detailed reports are prepared, followed by further big data analysis.

This study used descriptive statistics to highlight the need for further analysis of cases with a length of stay (LoS) over one day. Crosstabulation and chi-squared tests confirmed a significant relationship between DRG cases with different LoS values. Additionally, the Kruskal–Wallis H test assessed how DRG changes affect hospital revenue, identifying significant differences among groups. The purpose of these methods was to identify significant patterns and impacts related to DRG coding changes, thereby guiding improvements in coding practices and hospital revenue management. The final stage is the action phase, where all analyses and validation of the data lead to the adoption of actions, categorized into three categories: training, rules and restrictions, and prediction models. All of these steps contribute to the improvement of coding, documentation, and quality of care, which is the final step before the process begins again with the auditing phase.

### 2.3. Final Reporting and Reassessment

The final report included a comprehensive analysis of the data obtained from the digital audit platform. The analysis was conducted in two phases: the main audit project phase and a second phase focused on rechecking a subset of the cases. This second phase aimed to oversee the auditors’ work. Specifically, 10% of the previously audited cases were rechecked by KETEKNY to ascertain the success of the action and verify the auditors’ accuracy.

### 2.4. Audit Results and Categorization

The audit results were analyzed and categorized within the digital audit platform. The findings were grouped into key categories: cases that were fully correct with no issues identified, cases requiring coding changes—such as incomplete or incorrect coding of diagnoses (including errors in primary or secondary diagnosis coding) or medical procedures—and cases with observations. The latter included issues like incomplete input documentation, incomplete files, insufficient coding documentation, missing practices, absent secondary diagnoses, missing information notes, missing medical procedures, and incomplete patient histories. Additional findings that did not fit into these categories were also noted.

## 3. Results

The results are presented in three distinct sections to enhance clarity.

### 3.1. Initial Audit Findings and Coding Accuracy

**Figure 3:**
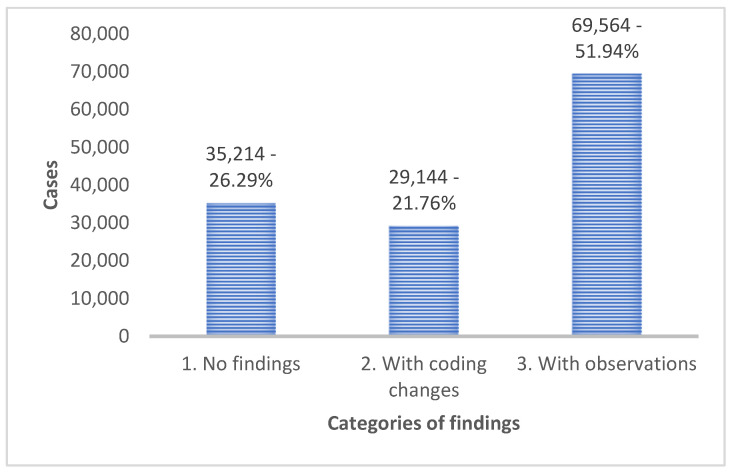
Audit results of all cases based on three main categories of findings.

A complete analysis of a sample coded by the coders of the Hospitals of the Crete Healthcare Region indicated that 26.29% of the sample had no coding errors and was fully correct, 51.94% of the cases had observations, and 21.76% of the overall sample required coding changes in the initial phase of the audit (Figure 3). An interesting finding was that only 9022 out of the 133,922 analyzed and audited cases (6.7%) ultimately had their DRG code changed. The remaining cases (93.3%) had their original DRG codes unchanged. Of the cases changed, 13.91% remained within the same DRG group, but were assigned a code for more complex cases.

To identify discrepancies across various coding categories, the entire sample was re-analyzed. The results indicated that 6.61% of the cases had a different primary diagnosis, 27.63% had a different secondary diagnosis, 17.62% had a different medical procedure, and 0.75% had a different category regarding the pathological and surgical sector. Finally, 29.19% of the incidents reviewed were labeled as “Incomplete File”, whereas 30.70% had an absence of medical notice and practical surgery.

Regarding the rechecks phase, out of 46,762 rechecks reviewed by the project team’s medical examiners, it was found that in 21.8% of the sample there were no findings and the coding was deemed correct, whereas 52.1% of the incidences resulted in observations, and 26.1% resulted in coding changes. Furthermore, 6.8% of the cases had a different primary diagnosis, 23.2% had a different secondary diagnosis, and 18.3% had a different medical procedure. Finally, 32.47% of the incidents reviewed were labeled as “Incomplete File”, while 39.27% of the cases had an absence of medical notice and practical surgery.

### 3.2. Impact of Length of Stay on DRG Code Changes

However, the analysis excluded cases with a one-day LoS, which are typically less complex, simplifying medical coding for primary and secondary diagnoses and medical procedures. A second analysis was conducted for cases with a LoS longer than one day. Among these cases, which represent 41.82% (56,009) of the total sample, 19.26% had no coding errors, 37.75% had some observations requiring further investigation, and the remaining 42.99% required coding changes.

**Figure 4:**
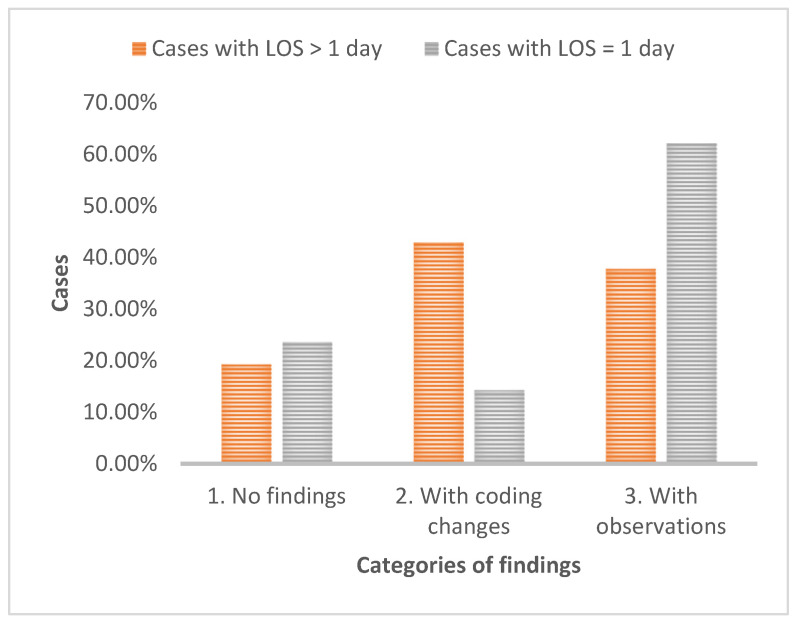
Percentage of status cases with LoS > 1 day vs. cases with LoS = 1.

The analysis revealed that cases with a LoS exceeding one day were nearly twice as likely to require coding changes, compared to total cases, and three times more than the ones with LoS equal to one (Figure 4). The analysis revealed that 12.35% of DRG codes required changes. This is an important finding, as it represents a nearly two-fold increase compared to the overall case analysis. Among the changed codes, 16.72% remained within the same DRG group, possibly due to minor coding adjustments. More significantly, 90.92% of the DRGs which remained in the group resulted in upgrades to higher-valued DRGs, suggesting increased resource utilization or case complexity. The remaining 9.75% of changes involved downgrades to lower-valued DRGs, which may indicate coding errors that initially attributed a higher acuity level than was accurate.

**Figure 5:**
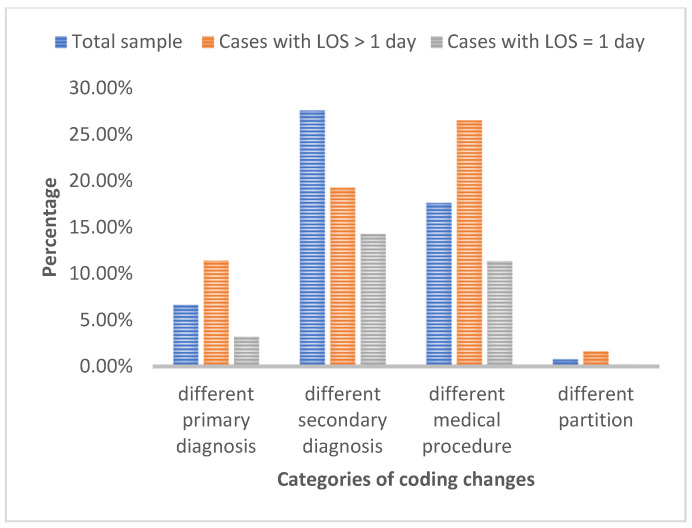
Percentage of status of total cases vs. cases with LoS > 1 day vs. cases with LoS = 1.

A broader analysis of the results follows. An important finding is the presence of discrepancies in coding categories across cases in comparison with the total sample (Figure 5). Notably, 11.40% of cases had a different primary diagnosis, 19.26% had a different secondary diagnosis, and a substantial 26.54% involved a different medical procedure code. Interestingly, discrepancies within the pathological and surgical partition category were much lower at only 1.60%. The above finding highlights the importance of auditing the primary diagnosis and the medical procedure of the cases with LoS longer than one day.

To assess whether there is a significant association between the need for DRG code changes and the duration of the patient stay in the hospital, we conducted a chi-square test of independence. Length of stay is a critical factor because it often indicates the complexity or severity of a patient’s condition, which can influence the accuracy and appropriateness of the initial DRG coding. This relationship was further illustrated using crosstabulation to provide a visual representation of the frequency distribution of the two variables (Table 1).

To test the hypothesis that LoS has a significant influence on medical coding, particularly leading to cases where DRG codes require changes, a Pearson chi-square test was conducted to assess the association between the LoS (categorized as one day versus more than one day) and the occurrence of a DRG code changes. The results (Table 2) revealed a chi-square value of 4824.574 (*p* < 0.001), implying a very strong likelihood that LoS influences the need for modifications in DRG coding. Specifically, when LoS extends beyond one day, the probability of requiring a DRG code change increases significantly. 

### 3.3. Hospital-Specific Variations in DRG Code Reclassification

Building on the initial analysis, we extended our investigation to explore how the relationship between a LoS exceeding one day and the frequency of DRG code changes varies across different hospitals. By doing so, we aimed to understand whether this relationship is consistent across various healthcare institutions or if there are notable differences that could indicate hospital-specific factors influencing DRG coding practices. Table 3 presents the results of this analysis, which involved comparing the incidence of DRG code changes for patients with a LoS exceeding one day across multiple hospitals.

The analysis concludes that there is a statistically significant association between hospitals and the frequency of DRG code changes, as demonstrated in Table 4. This finding indicates that the likelihood of DRG code modifications is not uniform across all hospitals, but rather varies significantly from one institution to another.

Results also indicated that a significant proportion of DRGs were reclassified by the auditors into different DRGs, with most being recoded to higher-cost-weighted DRGs. This reclassification could lead to a substantial increase in hospital revenue. To investigate how hospital revenue was affected, we examined whether the DRG assignment after the audit improved or worsened revenue to the same extent for all hospitals. The ordinal DRG change data (unchanged, improved, worsened) was categorized for each hospital and compared using non-parametric tests.

**Figure 6:**
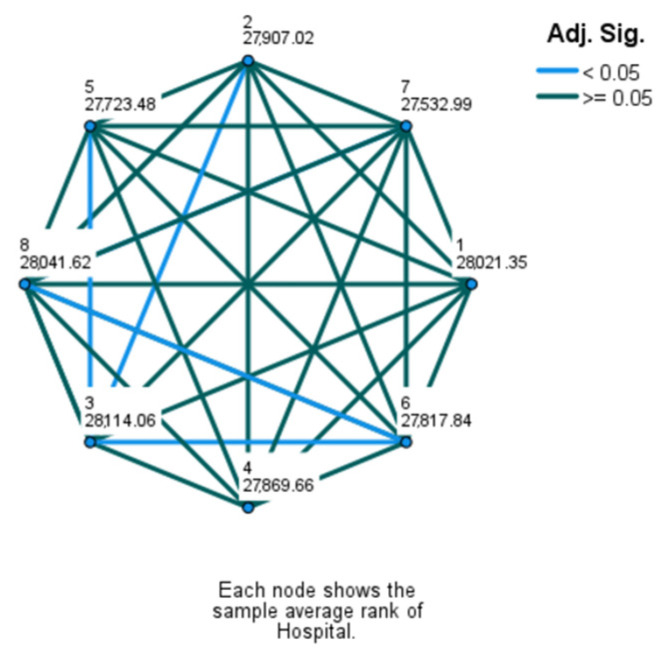
Pairwise comparisons of hospitals.

The Kruskal–Wallis H test revealed a statistically significant difference (χ^2^(2) = 40.005, *p* < 0.001) in how DRG changes impacted revenue across hospitals. The mean rank scores were as follows: Hospital 1 (28,021.35), Hospital 2 (27,907.02), Hospital 3 (28,114.06), Hospital 4 (27,869.66), Hospital 5 (27,723.48), Hospital 6 (27,817.84), Hospital 7 (27,532.99), and Hospital 8 (28,041.62). Since the null hypothesis of the Kruskal–Wallis test was rejected, indicating an overall difference, Dunn’s test for non-parametric pairwise multiple comparisons was conducted to identify which hospitals have statistically different outcomes in revenue change (results are illustrated on Figure 6). This could imply that certain hospitals might need to adjust their coding practices or audit procedures to optimize revenue, or that there are systemic differences in how patient care is coded across hospitals.

In cases where the length of stay (LoS) exceeded one day, the primary errors were linked to various coding issues, such as the omission of secondary diagnoses and medical procedures that should have been documented, as well as the use of overly general codes. Analyzing and comparing cases with an extended LoS highlights the importance of targeted analysis and actions tailored to different patient segments, as the results can vary significantly when viewed from different perspectives. Beyond LoS, further analysis could be conducted by hospital, clinic, major diagnostic category, admission type, and other relevant factors to gain a deeper understanding of these discrepancies.

## 4. Discussion

The findings from this study underscore the importance of considering LoS when evaluating the accuracy of DRG coding, as longer hospital stays often reflect more complex or severe cases, which may not be adequately captured by the initial DRG code assignment. The analysis also revealed that while some hospitals exhibit a strong correlation between extended LoS and subsequent DRG code changes, others show a weaker or even negligible association. These differences suggest that factors such as hospital size, available resources, coding procedures, and the complexity of cases managed by the hospital could influence the likelihood of DRG code revisions. For instance, larger hospitals or those handling more complex cases may have more stringent auditing processes, leading to a higher incidence of DRG code changes for longer stays [15]. Overall, this highlights the importance of considering institutional variability when evaluating DRG coding practices. Understanding these differences can help in developing tailored strategies for improving coding accuracy and consistency across various healthcare settings.

The provision of medical coding control and consulting services can be instrumental in the proper implementation of the DRG classification system, by fostering accurate medical coding and ensuring transparent allocation of resources within hospitals. The main actions that can be regulated and adopted for the proper implementation of the DRG system might include correct and complete entry of all medical data into the electronic medical record to ultimately retrieve correct information, as well as correct coding of all necessary medical information. By classifying patients into DRGs based on their condition, hospitals can improve the accuracy of medical records and ensure they are receiving appropriate reimbursement, ultimately leading to higher quality healthcare [16].

In Greece, the DRG system has been connected to the majority of public hospitals’ digital patient files. This, in itself, is an opportunity for the coding software to be further developed to do more than suggest codes or prevent coder errors through pre-programmed rules and edits based on official coding manuals. It can also be developed to check the assigned diagnosis and procedure codes for accuracy [5]. Initial rules and restrictions have been implemented, such as preventing coders from making errors based on age/sex, and prohibiting assistance in the coding process using “balloons” (which likely refer to prompts or suggestions). It can also be further developed to identify potential coding errors. This could involve a coding and validation process that checks for missing codes or incorrect code combinations.

Additionally, by applying coding logic, the software could highlight cases where a certain procedure would not typically be performed for a specific diagnosis, prompting the coder to review the case in real-time. A coding system like this could be implemented by checking the medical materials used during a hospitalization and the invasive medical procedures performed. For example, if medical materials are used, but no corresponding medical procedure is coded, the software would prevent the case submission until the medical procedure is documented. Furthermore, analyzing the data can lead to more sophisticated methods for adding value to coding quality. This includes filtering data, adding rules and improving the user experience of medical data records. For instance, adding DRG information to the home page of medical records can provide a solid foundation for both a comprehensive evaluation of DRG effectiveness and improvements in overall medical quality [17].

Our results suggest variation in DRG code modifications across hospitals. We did not investigate specific factors such as coding practices, resources, patient demographics, or case complexity, although the literature indicates that these factors often influence DRG code modifications [18,19]. Hospitals with robust auditing and coding processes tend to have more frequent code adjustments, whereas those with fewer resources show fewer changes. This underscores the need for tailored interventions to enhance DRG coding accuracy at the hospital level. Customizing actions—such as improving training, auditing, and resource allocation—can ensure accurate DRG coding, leading to better reimbursement and healthcare quality.

Deep learning and artificial intelligence techniques can be employed to develop accurate DRG prediction models that identify primary and secondary diagnoses and medical procedures. This platform is a crucial part of the data analysis framework for DRG prediction. It uses deep supervised learning to evaluate the database’s quality and completeness, and ensures the creation of labeled datasets for both training and testing. These models use a loss function to measure accuracy and adjust their weights to minimize errors [20,21]. Research indicates that deep learning models can achieve high accuracy in predicting primary diagnoses for DRGs. Implementing such an automated DRG coding system could reduce incorrect coding, potentially increase hospital revenue, improve resource allocation, and enhance overall hospital performance [22,23].

Another important action that can be adopted is the organization of post-audit training of coders (physicians) who must know what findings were made, be aware of miscoding, and adhere to the rules of DRG coding. KETEKNY has already organized the first presentation of this finding and plans to further adapt it. In the Greek DRG system, physicians currently handle the coding of cases. While this allows them to describe each incident in greater detail and more consistently, surveys indicate that many hospitals are hesitant to transition entirely to specialist coders [24]. Customized and targeted training is suggested, with material that is enriched by the results of the audits with coding quality [25]. These trainings can be highly targeted and adaptive to small-group training when necessary. Sometimes post-test training is effective at a large-group level, but sometimes it still needs to be one-on-one to maximize effectiveness.

A notable limitation of this study, which should be taken into consideration, was the scarcity of healthcare professionals with specialized expertise in code auditing, which posed a significant challenge during the auditing process. Additionally, since the hospitals were in the early stages of DRG system implementation, their coding practices were less mature. This necessitated increased guidance and support for hospital staff, resulting in a greater effort and time investment than initially anticipated.

## 5. Conclusions

This project aims to establish a foundation for future initiatives, including a permanent sample control process through the KETEKNY platform. It will involve selecting cases from all Greek hospitals based on various criteria to ensure accurate hospitalization data recording in medical files and the Grouper system, aligning cases with the correct DRG. A proposed strategy is to adjust audit frequency based on past performance. Hospitals with better coding records would face fewer audits, while those with poorer performance would be audited more often. This approach could encourage competition and improvement among hospitals. Additionally, there is an increasing need for specialized solutions tailored to individual hospitals.

The more specialized and unique the hospital’s spatial and organizational characteristics, the greater the need for the implementation of custom tools. Currently, the KETEKNY platform connects directly with hospitals’ electronic patient files. It complies with GDPR regulations and aims to extend this direct connection to the rest of the Greek healthcare regions. The concurrent implementation of all the above actions, along with the recent assignment of responsibility for the Gr-DRG system to an independent, self-governing body, can achieve a clear strategic objective: optimal resource allocation among all hospital care providers in Greece, both in the public and private sectors. This approach would also support the establishment of clear standards and practical guidelines, with the necessary legal and policy support.

## Figures and Tables

**Table 1:** Crosstabulation—DRG change vs. LoS (% of total).

Length of Stay				
		Cases with LoS > 1 Day	Cases with LoS = 1 Day	Total
DRG Change	No	36.7%	56.6%	93.3%
	Yes	5.2%	1.6%	6.7%
Total		41.8%	58.2%	100.0%

**Table 2:** Chi-square tests for checking association of LoS = 1 and LoS > 1 regarding DRG code changes.

	Value	Df	Asymptotic Sig. (2-Sided)	Exact Sig. (2-Sided)	Exact Sig. (1-Sided)
Pearson Chi-Square	4824.574 ^a^	1	0.000		
Continuity Correction ^b^	4823.039	1	0.000		
Likelihood Ratio	4861.607	1	0.000		
Fisher’s Exact Test				0.000	0.000
Linear-by-Linear Association	4824.537	1	0.000		
N of Valid Cases	133,922				

^a^ 0 cells (0.0%) have expected count less than 5. The minimum expected count is 3773.19; ^b^ Computed only for a 2 × 2 table.

**Table 3:** Crosstabulation—hospital vs. DRG change (% of total).

		DRG Change		
		No	Yes	Total
Hospital	Hospital 3	21.2%	3.5%	24.7%
	Hospital 2	15.2%	1.9%	17.2%
	Hospital 4	2.3%	0.2%	2.5%
	Hospital 5	2.5%	0.1%	2.6%
	Hospital 6	6.2%	0.7%	6.8%
	Hospital 1	2.9%	0.4%	3.4%
	Hospital 7	0.5%	0.0%	0.6%
	Hospital 8	36.8%	5.5%	42.2%
Total		87.7%	12.3%	100.0%

**Table 4:** Chi-square tests for checking the association.

	Value	df	Asymptotic Sig. (2-Sided)
Pearson Chi-Square	227.591 ^a^	7	<0.001
Likelihood Ratio	270.451	7	<0.001
N of Valid Cases	56,009		

^a^ 0 cells (0.0%) have expected count less than 5. The minimum expected count is 39.14.

## Data Availability

The data presented in this study are available on request from the corresponding author.
